# Using low-cost sensors to assess common air pollution sources across multiple residences

**DOI:** 10.1038/s41598-025-85985-1

**Published:** 2025-01-13

**Authors:** Catrin J. Rathbone, Dimitrios Bousiotis, Owain G. Rose, Francis D. Pope

**Affiliations:** School of Geography, Earth and Environmental Sciences, University of Birmingham, Edgbaston, Birmingham, B15 2TT UK

**Keywords:** Environmental impact, Environmental monitoring

## Abstract

The rapid development of low-cost sensors provides the opportunity to greatly advance the scope and extent of monitoring of indoor air pollution. In this study, calibrated particle matter (PM) sensors and a non-negative matrix factorisation (NMF) source apportionment technique are used to investigate PM concentrations and source contributions across three households in an urban residential area. The NMF is applied to combined data from all houses to generate source profiles that can be used to understand how PM source characteristics are similar or differ between different households in the same urban area. PM_2.5_ and PM_10_ concentrations in all three houses were greater, more variable, and significantly different to ambient concentrations recorded at a nearby ambient monitoring site. Concentrations were also significantly different between houses, with the World Health Organisation 24-h guideline limits for PM_2.5_ breached in one household. The applied methodology was highly successful at modelling concentrations for all the houses (R^2^ $\ge$ 0.983), finding that across the houses the I/O (indoor to outdoor sources ratio) was the lowest for PM_1_ (down to 0.08), and greatest for PM_10_ (up to 4.93). Whilst the sources could not be clearly distinguished further than being outdoors or indoors, the methodology provides clear insights to source variability within and between the monitored houses. These results highlight the importance of monitoring indoor air pollution to improve pollution exposure estimates, as whilst people may live in areas with acceptable ambient air quality, they can be exposed to unhealthy concentrations in their own homes. This method may be applied in future studies for extended periods to investigate the influence of source seasonality on PM concentrations or scaled up to investigate source variability across larger geographical areas.

## Introduction

Indoor air quality represents a significant proportion of human pollution exposure, with an increasing amount of time being spent indoors (80–90%)^1,2^, and a rise in home-based and hybrid working since the COVID-19 pandemic^3^. Globally, household air pollution is an important health determinant, responsible for 3.2 million premature deaths in 2020^4^. Additionally, there is increasing evidence indicating the negative impacts of indoor air quality upon cognitive functioning^5,6^. With more time being spent indoors and working from home, understanding the factors that affect air quality within households is becoming increasingly important.

Particulate matter (PM) are solid or liquid droplet particles comprised of a range of chemical compounds and shapes, typically classified by size (e.g. PM_10_, aerodynamic diameter < 10 μm) due to the evidence of size upon health effects^7^. Indoor PM pollution can be from primary particles emitted by indoor sources, resuspension of particles, ambient aerosols entering from outdoors, or be secondary particles which form indoors^8^. Exposure to fine PM (PM_2.5_) is of particular concern as once inhaled, smaller particles are able to penetrate deeper into the respiratory system where they may enter the blood stream and be transported to other organs^9^. Resultingly, PM_2.5_ exposure is one of the leading environmental health risk factors, causing millions of annual premature deaths and numerous detrimental health impacts^10,11^. Ambient PM_2.5,_ along with PM_10_, are the particulate matter size fractions currently regulated, and in 2021 the World Health Organisation updated their recommended guideline limits in response to growing evidence of the adverse health effects of exposures^12^.

Across indoor air pollution studies, the presence and movement of people has been found to be important for PM_10_ concentrations^13^, while cooking, smoking and heating are major sources of PM_2.5_^14^. For ultrafine particles (PM_0.1_) outdoor concentrations, building characteristics, air exchange rates and meteorological parameters are important^14^. Research into ambient pollution building infiltration has found buildings to be ‘incomplete filters’ with outdoor particles able to enter through open doors and windows, and smaller ones penetrating gaps in building facades^15^. Cooking has also been extensively studied, regularly finding cooking activities associated with high PM peaks, particularly for ultrafine particles, and high exposure levels during post-cooking decay phases^16,17^. These findings are important, as they show that the larger PM particles are typically generated by the presence of humans and their activities, however humans have less control over their exposure to smaller particle sizes.

Despite these findings, there is generally a less comprehensive understanding of indoor air quality compared to ambient air quality, for which many countries undertake regulatory monitoring and have established pollution standards and legal limits^18^. Establishing effective policies and regulations requires accurate data evidence of pollutant levels and their sources^19^. Achieving this for indoor air pollution is challenging due to the heterogeneity of indoor environments, which are affected by both indoor pollution sources, the infiltration of outdoor pollution sources, and factors such as ventilation parameters, building characteristics and occupant behaviour impacts^20,21^. To improve the accuracy of indoor air quality exposure estimates which often rely on outdoor monitoring network data, better quantification of the influence of both indoor and outdoor pollution sources is needed^21^. Monitoring, quantifying and modelling indoor pollution to the same extent as has been achieved for ambient air pollution is difficult due to the resource intensity required, associated costs, and the ethical and practical challenges^20^; as such, understanding the heterogeneity of indoor air pollution is a continuing challenge.

Development of low-cost sensors (LCS) which are smaller, cheaper and less power intensive offer the opportunity to expand indoor air quality monitoring by increasing the density of measurements able to be made^22^. An expansion of studies deploying LCS indoors and outdoors at large scales and as networks has been seen^23,24^ with improved pollution exposure estimates resulting from the increased spatial availability of data^11,15^. These studies consistently show that higher activity levels lead to increased particulate matter (PM) concentrations and peaks associated with cooking and periods of higher indoor movement^23,24^. Additionally, an investigation into indoor air quality in university residences in Canada found that indoor PM_2.5_ measurements correlated with outdoor concentrations, but that this was disturbed when indoor sources dominated^21^.

Whilst indoor source apportionment is less frequently undertaken than ambient source apportionment^25^, a recent paper by Bousiotis et al.^13^ was the first to successfully apply source apportionment methods to LCS data in an indoor setting. PM concentrations and sources were compared between different indoor microenvironments within a family home. The study by Bousiotis et al.^13^ found that that average PM_2.5_ and PM_10_ concentrations were highest in the bedroom where there were more soft furnishings, but biggest concentration peaks occurred in the kitchen during cooking activities. The source apportionment work undertaken in the study showed outdoor sources were dominant for all PM size fractions, but most important for PM_1_, with concentrations highest in the office which had the greatest natural ventilation rates.

Recent studies have demonstrated the potential of LCS to improve the accuracy of pollution exposure models. The development of LCS source apportionment techniques provides the tools to better understand the sources and factors influencing this exposure.

As well as having been shown to improve PM pollution exposure estimates, other studies have found LCS can correlate well with reference instruments, and detect local pollution hotspots though increased data spatial variability^26,27^. However, LCS have numerous associated trade-offs, being less sensitive, precise and chemically specific than traditional reference instruments, as well as issues of sensor failures^22,26^. Additionally, they are often affected by atmospheric conditions, need regular careful calibration and post-processing of the data – without which they can perform poorly^13,24,28^. For LCS such as Optical Particle Counters (OPCs) developed for PM monitoring which rely upon light-scattering principles, high relative humidity (RH) can be problematic^29^. At high RH, particles can uptake water, growing in size and mass which alters the light scattering of the particles, and influences the particle refractive index, shape and density^28^. If hygroscopic material is present, high RH can result in an overestimate of PM masses, giving misleading results and potentially unnecessary interventions being taken^30^. Conversely, lower limits of particle size detections of these LCS mean there is a potential to not detect a significant proportion of mass if lots of particles are smaller than this size^28^. This can result in false impressions of acceptable air quality^30^.

This study investigates the variability of PM concentrations and sources using LCS data across multiple households within the same residential area of Selly Oak, Birmingham. In doing so, it expands upon the recently developed LCS source apportionment work, to see how the technique performs across numerous households within the same neighbourhood. This gives an indication of the heterogeneity of PM and source contributions at a residential scale, to build an understanding of the data resolution needed to generate models for source exposure estimates for a given urban area. To achieve this, Alphasense OPC-N3s are used to gather PM data, including particle number size distribution (PNSD), in three student households, and participant activity data recorded in time activity diaries, across a 2-week monitoring campaign during March. Meteorological and ambient PM data are obtained from an established monitoring station approximately 1.5km north of the study area. NMF source apportionment is applied, and results analysed in conjunction with activity data, meteorological conditions and background concentrations to identify sources detected across the houses, and estimate their effect upon PM concentrations.

## Results

### Calibration

Following the 2-day indoor collocation with the TSI-3330 reference instrument, independent calibrations were performed for each OPC for the PM_1_, PM_2.5_ and PM_10_ size fractions. Before calibration, the three OPCs were in good agreement with the TSI, with a Pearson’s correlation coefficient of *r* $\ge$ 0.799 for all PM size fractions (correlation plots found in supplementary material Fig. S1. High linear agreement pre-calibration between the LCS and TSI occurred due to the absence of high relative humidity in the indoor residential environment that is often seen outdoors which lead to PM mass concentration overestimates. To calibrate the OPCs, a linear calibration with zero intercept was used as the most appropriate calibration method. The choice of zero intercept disallows negative values in the calibrated data set. This was done since negative PM concentrations are not physically realistic. Agreement between the LCS and TSI remained high after calibration (*r* $\ge$ 0.799).

### Study conditions

During the monitoring campaign, ambient background PM_2.5_ and PM_10_ concentrations and standard deviation were 4.82 (± 3.28) μg m^−3^ and 6.93 (± 4.36) μg m^−3^ respectively. The outdoor conditions were cool with temperatures averaging 6.8 (± 4.6) °C, and relative humidity of 87.3 (± 10.9) %, with south-westerly winds 71% of the time.

Throughout the campaign, the houses were mostly part-occupied, with H1 empty only 10% of the time, H2 21% and H3 13%. The monitored bedrooms themselves were unoccupied more often: 54% of the time at H1, 65% at H2, 67% in H3. With occupants being students, the timings of empty periods were variable day-to-day and between the houses. A 24-h period of the respective bedrooms being empty occurred in each house at least once throughout the monitoring campaign: H1 (16:00 March 14–12:30 March 16 & 00:00 March 18–22:00 March 19), H2 (20:00 March 11–20:00 March 12) and H3 (10:00 March 8–16:15 March 9). Similar cooking activity levels occurred in each house (6.6–7.7% of the time), with H3 generally making use of an extractor fan, but little to no ventilation occurring in H1 and H2. As with occupancy periods, cooking times and volumes were irregular throughout the campaign across the houses. Ventilation in the bedrooms themselves was also limited, largely owing to the cold time of year, with the bedroom window in H2 never opened, opened 3% of the time in H1, and 10% in H3.

### PM concentrations and variability

**Fig. 1:**
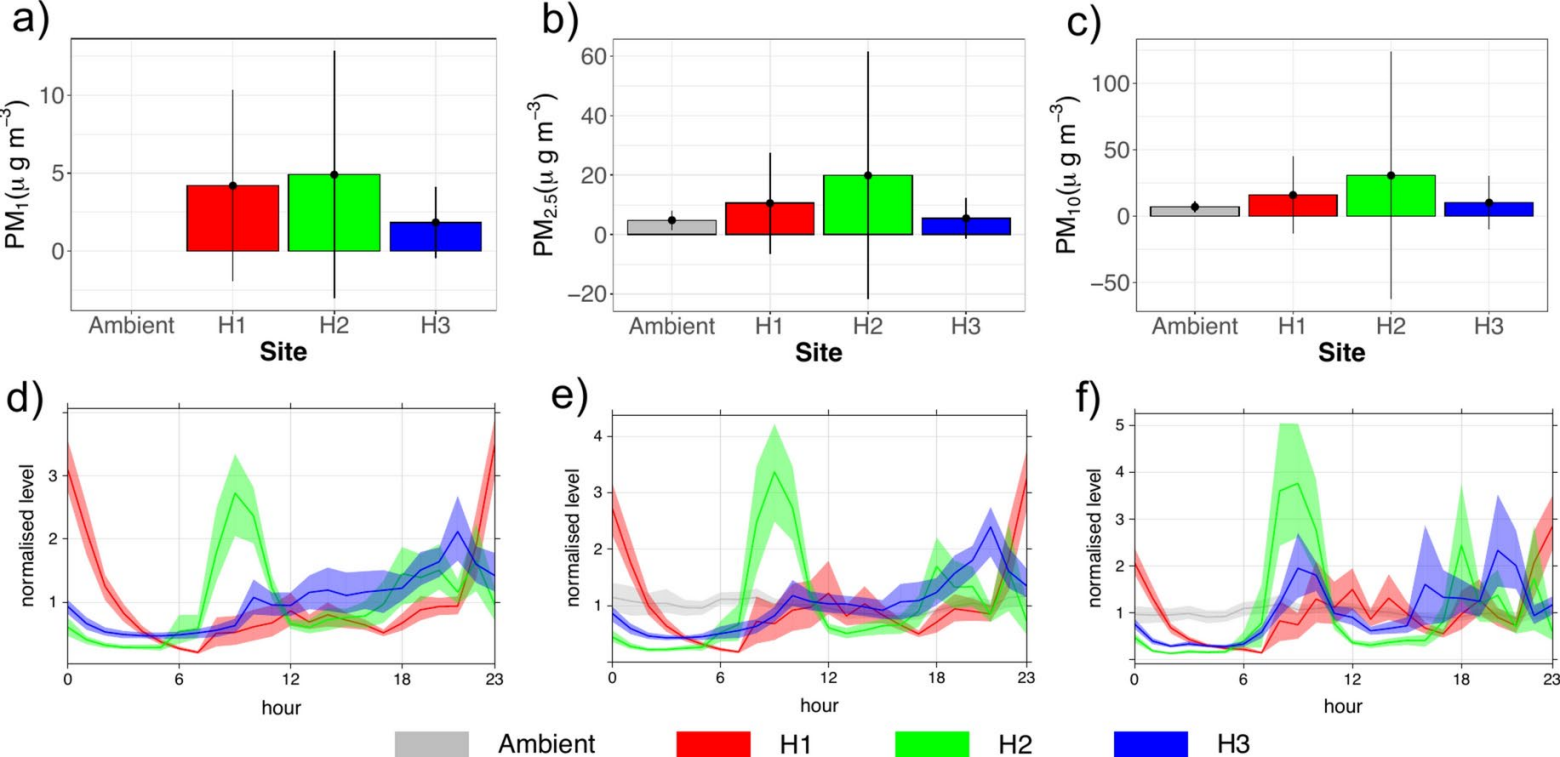
Mean concentrations with standard errors denoted by black vertical lines, at outdoor ambient monitoring site (grey), H1 (red), H2 (green) and H3 (blue) for PM_1_* (**a**), PM_2.5_ (**b**) and PM_10_ (**c**). Average normalised concentration diurnal variations at each site for PM_1_* (**d**), PM_2.5_ (**e**), and PM_10_ (**f**). *ambient PM_1_ data unavailable.

The average PM_1_, PM_2.5_ and PM_10_ concentrations and standard deviations, and their average diurnal variations recorded at each house and (where available) outdoors is shown in Fig. 1. Detailed summary statistics are found in the supplementary material (Table S1). For each PM size fraction, H2 recorded the highest concentrations, and H3 the lowest, with all houses PM_2.5_ and PM_10_ concentrations greater than ambient conditions. Kruskal–Wallis test result indicate the difference in PM concentration values between the sites are statistically significant at the 99.9% levels for PM_1_ (${\chi }^{2}$ = 606, df = 2, p < 0.001), PM_2.5_ (${\chi }^{2}$ = 591, df = 3, p < 0.001) and PM_10_ (${\chi }^{2}$ = 159, df = 3, p < 0.001). Post-hoc analysis results found there to be a statistically significant difference (p < 0.001) in PM concentrations between all site pairs for each PM size fraction, other than H1 and H2 for PM_1_. The non-significant difference between PM_1_ at H1 and H2 indicates this PM size fraction to be affected by a common variable, likely an outdoor source.

Distinct diurnal variations were observed in each house; morning and evening concentration peaks were observed in H2 across the PM size fractions, around typical times of activity in this bedroom. H3 also observed evening and (less distinct for PM_1_ and PM_2.5_) morning peaks. At H1, the diurnal profile sees distinct peaks across late evening hours, decaying in early morning hours across all PM size fractions.

Average 24-h mean PM_2.5_ and PM_10_ concentrations for each house and ambient conditions alongside WHO and UK ambient guideline 24-h limits are compared in Table 1. Average 24-h mean PM_10_ concentrations were below the WHO guideline (45.0 μg m^−3^) and UK guideline (50.0 μg m^−3^) for all sites, although both the WHO and UK 24-h limit for PM_10_ was exceeded on 2 days individually during the campaign in H2: March 15th and 17th, with both days over 55.0 μg m^−3^. The exceedance on March 15th is potentially due to vacuuming in the room which was noted in the activity diary. Ambient, H1 and H3 mean daily PM_2.5_ averages were below the guideline WHO guideline of 15.0 μg m^−3^ (with no UK 24-h PM_2.5_ guideline set), although it was exceeded on the 20th of March at H1, reaching 19.4 μg m^−3^. However, at H2 the average daily mean concentrations for PM_2.5_ exceeded the WHO guideline limit, and exceedances occurred on 9 individual days throughout the campaign—the highest on March 17th (35.4 μg m^−3^).

**Table 1:** Average 24-h mean PM_2.5_ and PM_10_ concentrations for each household and ambient over the two weeks given alongside the WHO and UK guideline limits, with the number of individual days of exceedances of each guideline given in brackets (WHO, UK).

	Average 24-h mean concentration (μg m^−3^)					
	WHO limit	UK limit	H1	H2	H3	Ambient
PM_2.5_	15.0	–	10.4 (1,–)	19.8 (9,–)	5.53 (0,–)	4.82 (0,–)
PM_10_	45.0	50.0	15.8 (0,0)	31.0 (2,2)	0,0)	6.98 (0,0)

### NMF analysis

NMF analysis was performed on a combined dataset of the three houses, to investigate how detected sources contributions to PM differed across the houses. Numerous solutions with different factor numbers were considered, and a 5-factor solution was selected as the optimum solution. NMF solutions with additional factors appeared to separate the five factors rather than provide additional separate sources.

PM concentrations were well estimated by the NMF, with high R^2^ values (0.983–0.999) between the measured and modelled PM concentrations for all PM sizes (correlation plots available in supplementary material Fig. S2). Accuracy was highest for PM_10_, showing the NMF was highly successful at identifying the sources which contributed to the larger PM particles.

**Fig. 2:**
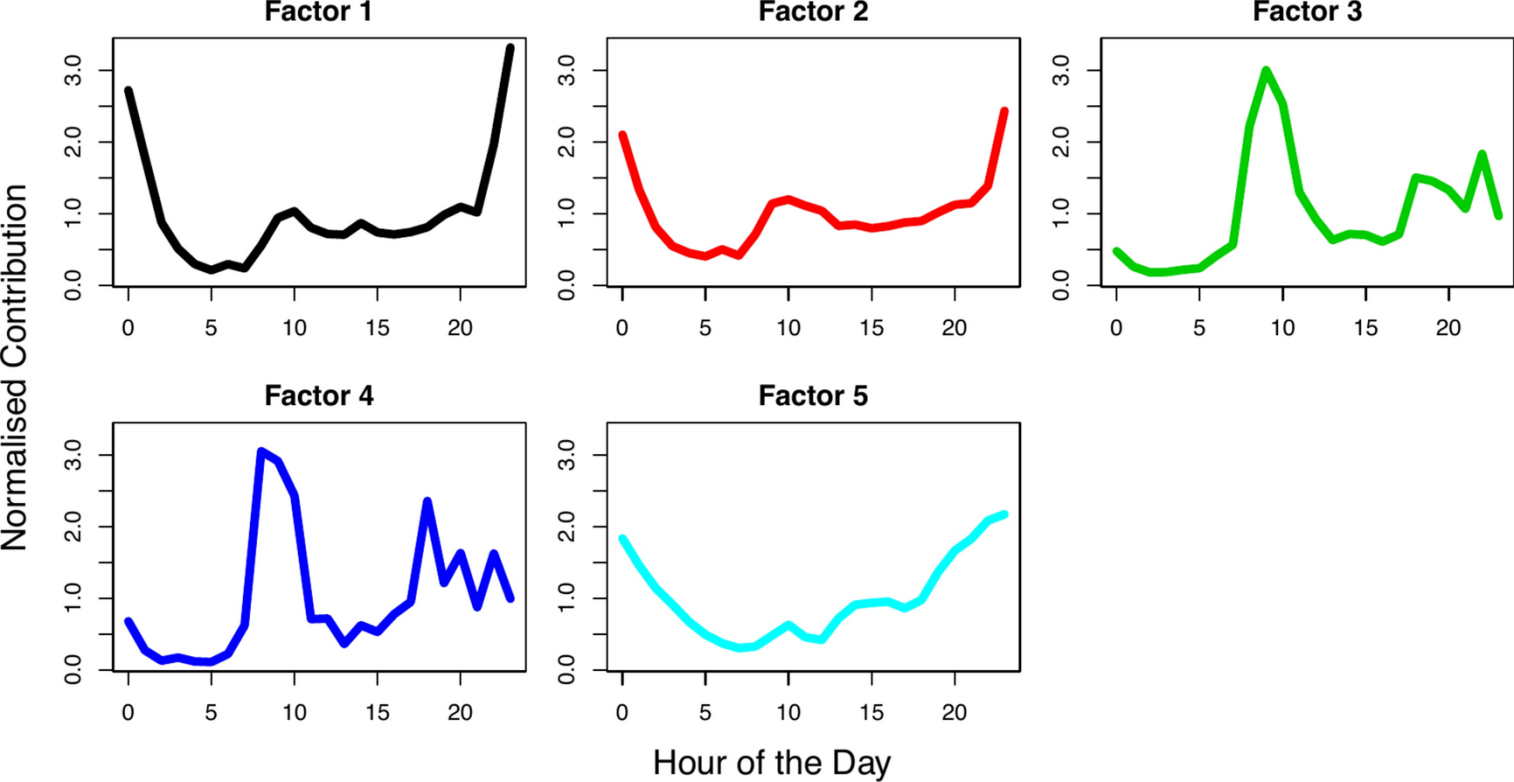
Average normalised factor G-contribution diurnal variation for each found factor returned from the 5-factor NMF source apportionment analysis.

The diurnal variation of each factor’s (F) average normalised G-contribution is given in Fig. 2 (average PNSD in supplementary material Fig. S3). F1, F2 and F5 have similar PNSD and diurnal contribution profiles, declining in early morning hours, and rising during the evening hours to highest contributions at midnight. Specifically, F1 and F2 also present a prominent secondary peak at ~ 10 a.m. F3 is characterised by count peaks at ~ 0.5 μm and 1.75 μm particle diameters, and distinct contribution peaks at ~ 9 a.m, and 6–10 p.m. F4 has a similar diurnal contribution profile to F3, however sees a multi-modal peak in counts for particle diameters between 1 and 7.5 μm. Whilst some pairs were found to have high Pearson’s correlation coefficients (Table S2), all factor combinations were tested and found significantly different to each other (p < 0.001).

**Fig. 3:**
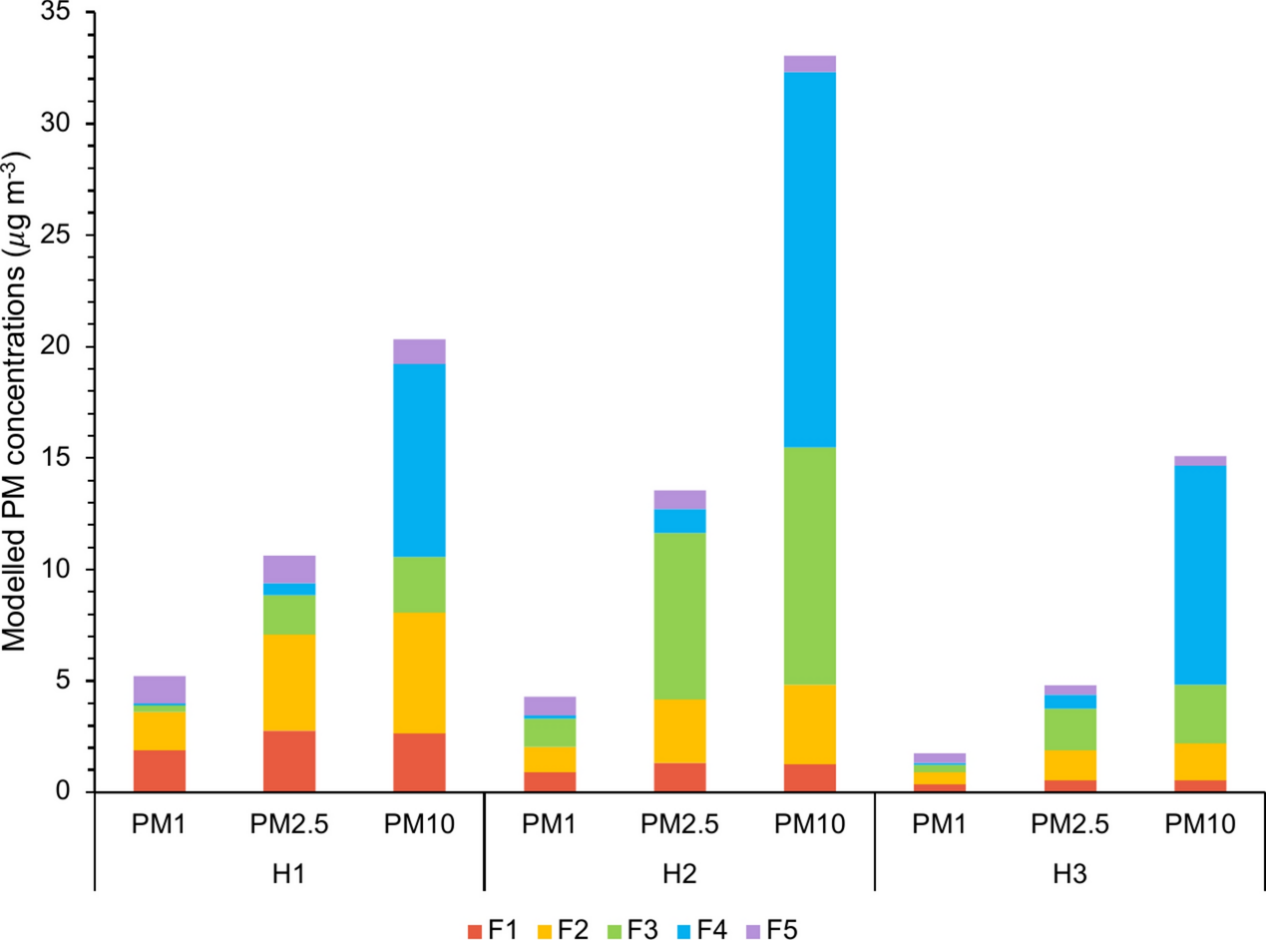
For each house, the average PM_1_, PM_2.5_ and PM_10_ concentrations modelled by the NMF source apportionment is shown, with a breakdown of the average contribution of each of the 5-factors to each PM size fraction at each house.

Each factors’ average G-contribution to the NMF modelled mean PM_1_, PM_2.5_ and PM_10_ concentrations in each house are shown in Fig. 3 (detailed data available in supplementary material Table S3). For PM_1_, F4 made the smallest contribution at each house, and conversely makes the greatest contribution to PM_10_ at each house (> 43% at each house). Interestingly, the biggest PM_1_ contributor is different between the houses, being F1, F3 and F2 for houses H1 to H3 accordingly. For PM_2.5_, F2 and F3 are also strong contributors, with F3 the biggest contributor at H2 and H3, whereas F2 dominates at H1.

**Fig. 4:**
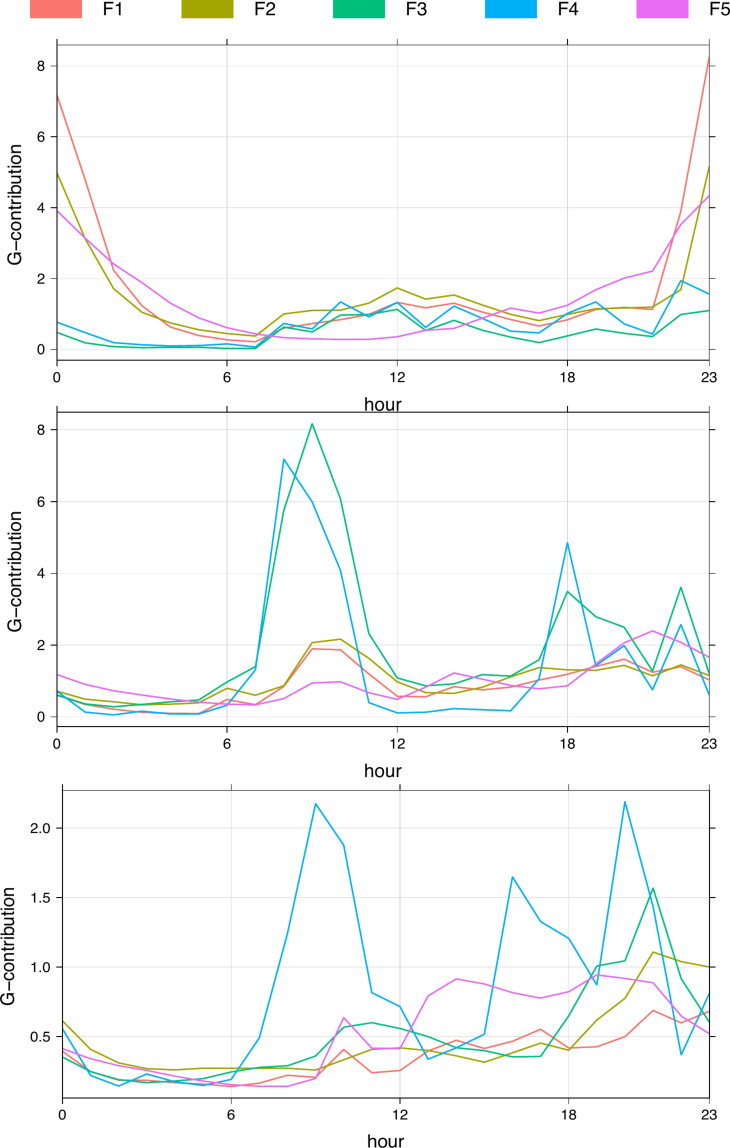
Average diurnal factor G-contribution for each household (**a**) H1, (**b**) H2, (**c**) H3.

Whilst factor average PM contributions are useful indicators of their influence upon PM concentrations, examination of the temporal variability of the factor contribution further indicates the sources associated with each factor. The average diurnal variation of the five factors G-contribution at each house is given in Fig. 4.

In comparison with the overall diurnal variation of the factors’ contributions (Fig. 2), Fig. 4 clearly shows that the distinct overnight peak for factors F1, F2 and F5 is characteristic to H1. The diurnal variations of F3, F4 and (in the case of H2 and H3) F5 are largely a reflection of the different activity times within each bedroom. In H2, F4 has a distinct contribution peak between 7–8 am, and about an hour later for F3 (Fig. 4b). Peaks for these factors are also seen in the evening ~ 6 pm and ~ 10 pm. These reflect typical times the H2 bedroom occupant would wake and get ready before leaving the house, and the times they would return and later go to bed. Similarly, F4 peaks around typical times of activity in the bedroom of H3 in the morning, late afternoon and evening, with smaller and delayed peaks in F3 also seen. At H1, there is no such distinct morning and evening peaks in F3 and F4, instead seeing numerous smaller peaks throughout the day, and almost zero contribution overnight. This reflects the more varied times in which the H1 occupant was in their bedroom—often leaving later in the day and sometimes returning throughout the day—compared to H2 and H3 where the bedrooms are typically empty mid-morning until early evening, as detailed in the activity diaries.

**Fig. 5:**
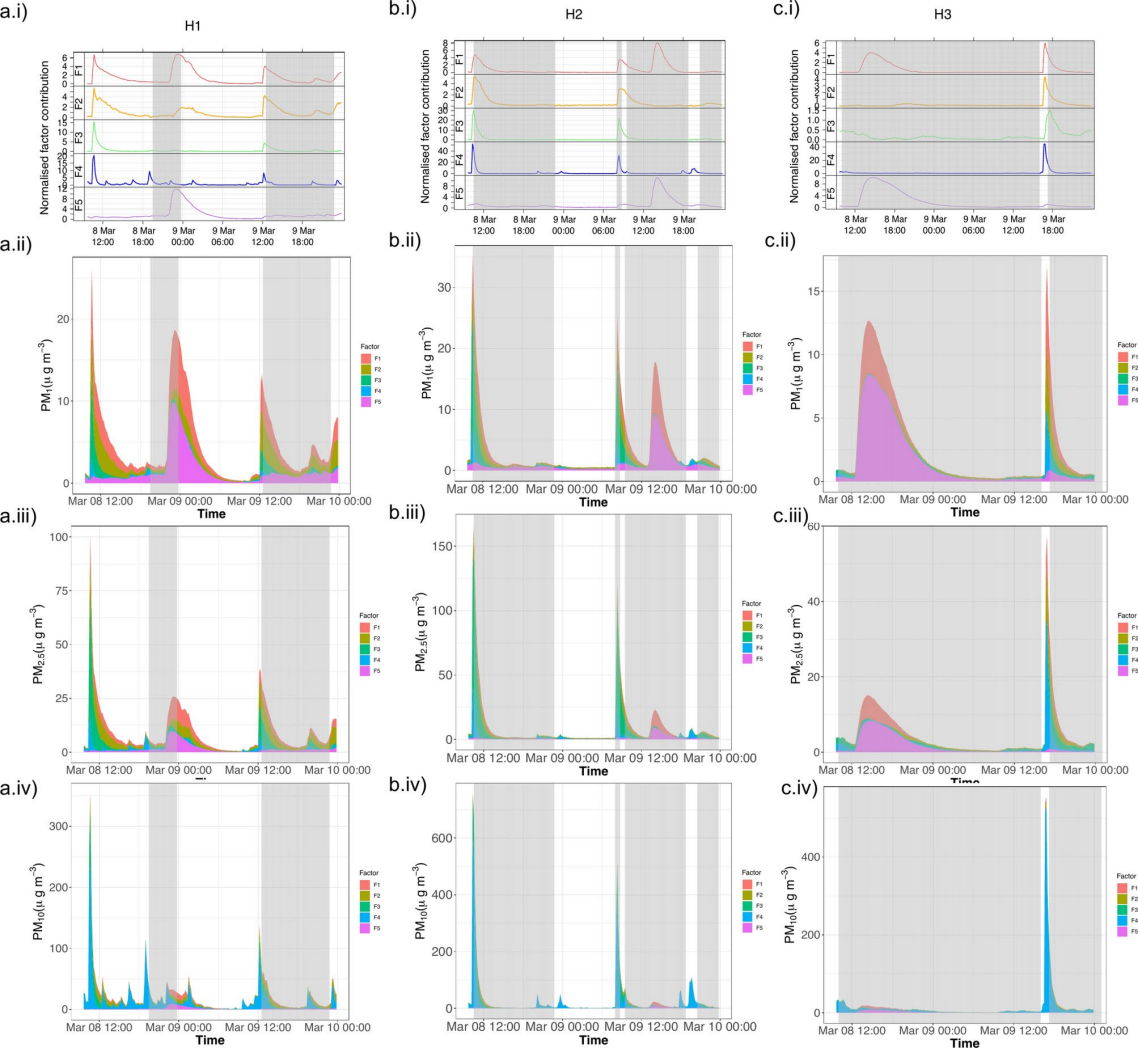
The normalised factor contribution temporal variability of each factor across the 8th & 9th of March (i), and each factors’ contribution to modelled PM concentrations for the same time period are given for PM_1_ (ii), PM_2.5_ (iii) and PM_10_ (iv), for H1 (**a**), H2 (**b**) and H3 (**c**). Grey shaded period indicates times the monitored bedrooms were unoccupied.

In Fig. 5, an example of the 5 factors’ contribution temporal variability and contributions to PM_1_, PM_2.5_ and PM_10_ modelled concentrations in each house are shown for the 8–9th of March. At H2 a distinct peak in factors F1, F2, F3 and F4 are seen ~ 10 a.m. on the 8th and ~ 8 a.m. on the 9th (Fig. 5b.i), corresponding to times the occupant got ready before leaving their room on these days. The F4 peak decays the fastest, followed by F3. During these events, F4 is the dominant contributor to PM_10_ concentrations, whereas F3 contributes most to PM_2.5_ and PM_1_ (Fig. 5bii-iv). The quicker peak and subsequent decay of F4 reflects its dominant contribution to the larger particle sizes; these heavier particles settle out faster, whereas F3 which is generally most dominant for the PM_2.5_ and PM_1_ particles reflect the slower settling times of these smaller particle sizes.

Whilst F3 and F4 have associations with activity times within the bedrooms, and each is associated with different particle size fraction decay behaviours, beyond being associated with times of bedroom occupancy, it is difficult to attribute particular activities to particular factors beyond indoor sources. Periods of cooking had no clear association to PM variability in the monitored rooms, nor did other activities noted by participants, though this can be justified by the distance of the kitchen to the bedrooms.

Factor F5 is an outdoor source. In Fig. 5, at each house a peak in PM_1_ concentrations occurs during times the bedrooms were unoccupied: ~ 11 p.m. March 8th at H1, ~ 2 p.m. March 9th at H2 and ~ 2.30 p.m. March 8th at H3. The relative G-contribution of F5 was highest and most variable at these times, indicating this to be an outdoor source, most influential when indoor activity and sources are absent. Peaks in larger PM size fractions were less pronounced at these unoccupied times, but also dominated by F5 contributions pointing to the limited infiltration of larger outdoor PM particle sizes.

Factor F1 is an interesting one. In H1, it appears to be associated with outdoor emissions, likely from the nearby restaurant. Within the other two houses, where such a distinct external source is missing, in many cases it seems to follow the other two indoor factors. Thus, it is plausible that this factor is associated with cooking activity, also supported by the low PM_2.5_ and PM_10_ contributions of this factor in H2 and H3, as cooking emissions are mostly associated with ultrafine (< 1 μm) particles^16^. For the following analysis, F1 is categorized as an outdoor factor, given the likely association with external commercial cooking seen at H1, in which it presented the highest contributions.

Overnight, variable peaks in F1 and F5 contributions were consistently seen exclusively at H1 most nights, often also accompanied by a F2 peak (with this encapsulated in the H1 factor diurnal variation in Fig. 4a). A notable example of this was on the evening of 12th of March when the occupant opened their bedroom window until midnight—it was noted in the time activity diary that the fast food kitchen vent located just outside their house was on during this time, and it was commented that this was the case most nights.

Given factors F3 and F4 as indoor sources and F1, F2 and F5 as outdoors, Table 2 gives the average indoor to outdoor source contribution ratios at each house, and as averaged across the three houses. Across the houses, indoor source contributions increase with increasing particle size, from PM_1_ to PM_10_. Ratios are < 1 for PM_1_ at all houses, and for PM_2.5_ at H1. Indoor sources dominate for PM_2.5_ at H2 and H3, and for PM_10_ at all houses. For all three PM size fractions, the ratios are lowest at H1 and below the average indoor source contributions. The I/O ratio is above average for all PM size fractions at H2 and H3.

**Table 2:** Indoor to outdoor source contribution ratios (I/O) for each PM size fraction at each house, and as an average across the three houses given.

	I/O ratio			
	H1	H2	H3	Average
PM_1_	0.08	0.50	0.31	0.30
PM_2.5_	0.28	1.71	1.07	1.02
PM_10_	1.22	4.93	4.82	3.66

The average PM concentrations and NMF modelled I/O ratios during periods where the monitored bedrooms were unoccupied for at least 24-h (see 2.2 for times) are summarised in Table 3. During the unoccupied periods, the average PM concentrations were lower than the modelled average across the monitoring campaign for all PM size fractions at all houses with the exception of PM_1_ at H3. The I/O ratios during the unoccupied periods were lower than average in all of the respective houses for PM_10_, and in H1 and H3 for PM_1_ and PM_2.5_ also.

**Table 3:** The mean PM concentrations and indoor: outdoor ratios (I/O) over the periods in which the monitored bedrooms were unoccupied for a minimum of 24-h.

Absent period	Average concentration (μg m^−3^)			Average I/O ratio		
	PM_1_	PM_2.5_	PM_10_	PM_1_	PM_2.5_	PM_10_
H1 (1)	3.03	5.06	6.86	0.02	0.06	0.34
H1 (2)	2.93	5.50	7.29	0.03	0.09	0.29
H2	3.62	12.79	26.67	0.64	1.90	4.15
H3	2.78	3.77	7.34	0.08	0.21	1.46

However, at H2 I/O ratios for PM_1_ and PM_2.5_ were slightly greater than average during the unoccupied period. Modelled PM concentrations and factor contributions at H2 showed the only major peak was associated with the occupant leaving the room. In all houses, during the periods the bedrooms were unoccupied, activity still occurred in other rooms, as the rest of the house was not unoccupied. The door of the monitored bedroom is assumed to be closed during periods of absence, thereby somewhat isolating the bedroom air from other household activities.

## Discussion

This study built-upon the recently developed similar LCS source apportionment methodology used for the first time in an indoor environment by Bousiotis et al.^13^ and expanded its application to estimate and compare PM concentrations and source contributions across multiple households.

### PM concentrations and variability

It was found that indoor PM_2.5_ and PM_10_ concentrations in all three houses were greater and more highly variable than ambient concentrations, and significantly different to ambient concentrations for all PM size fractions. Other than PM_10_ at H1, the measured PM concentrations were within the concentration ranges reported from a global review of indoor exposure studies for PM_2.5_ (1.7–428.6 μg m^−3^) and PM_10_ (11.0–1275.0 μg m^−3^)^14^. The reported ranges encapsulate the difficulty in comparing indoor air quality studies, owing to the indoor and outdoor conditions unique to each studies environment. Where studies have compared indoor and outdoor concentrations, some have found outdoor or ambient concentrations to be greatest^13,15,31,32^. However, higher indoor concentrations have also been found^24^, as was the case in this study: with average ambient PM concentrations relatively low (4.98 and 7.34 μg m^−3^ for PM_2.5_ and PM_10_) and high occupancy levels of each household both contributing to the greater indoor concentrations.

PM concentrations were also significantly different between the houses themselves, and average PM_1_, PM_2.5_ and PM_10_ concentrations at H2 were over double H3 concentrations, which saw the highest and lowest averages respectively. Additionally, whilst H3 and ambient conditions were below the WHO 24-h guideline limits for PM_2.5_ across the 2-weeks, H2 exceeded the PM_2.5_ guideline on nine individual days (64%). Resultingly, the 3–4 days annually allowed exceedances set by the WHO for daily PM_2.5_ concentrations have been exceeded at H2 in just the 2-week monitoring period. Exceedance of the PM_2.5_ guideline is particularly concerning due to the evidence of the detrimental health impacts of smaller particle inhalation^9^. This highlights the importance of indoor air quality exposure, and further supports the need to incorporate indoor concentrations to exposure estimates within literature^33^—showing that whilst living in an area which may meet ambient air quality standards, within their own homes’ individuals may be exposed to more unhealthy concentrations. These results also reflect the large heterogeneity in particulate matter concentrations that can be found across households within the same residential area in relatively close proximity, and of similar household nature. Thus, the data from just one household is likely an unreliable approximation for indoor concentrations across a residential area.

Although large differences in PM concentrations was found between H2 and H3, relatively similar diurnal variability was observed with distinct early morning and evening peaks. Dissimilarly, at H1 a unique overnight peak in PM concentrations was observed. NMF results indicate that these differences can be partly attributable to the different activity patterns between the monitored bedrooms, with H2 and H3 typically empty between mid-morning and early evening, whereas H1 was more variably occupied during the day. However, the adjacent fast-food kitchen vent and other fast-food outlet pollution source on Bristol Road also have an important role, operating late at night and impacting the overnight PM concentrations at H1.

### NMF performance

This study is the first to apply NMF source apportionment to indoor particulate matter data gathered using LCS across multiple households. The NMF was highly successful at modelling the PM concentrations across the houses, showing the model was able to quantify PM concentrations in the different houses based upon the identified factors, of which two were identified as indoors, and three as outdoor sources.

Despite the very different PM concentrations found at H2 and H3, the two indoor sources identified saw similar and consistent contribution peaks with clear associations to activity in the bedrooms, and each associated with different PM size fractions. Similar to findings of the PMF source apportionment applied to one household by Bousiotis et al.^13^, the methodology found that indoor factors were seen to respond to general activity in the bedrooms, but was unable to clearly distinguish specific individual activities or combinations.

At H1 the factor F1 consistently peaked overnight, as was also seen for the other two outdoor factor at this house. Such a distinct overnight peak was not seen at the other two houses. This difference in F1 behaviour between the different household environments indicates that whilst the same PNSD profile was detected across the three households, it is potentially corresponding to different sources in the different environments. A unique feature of H1 is its location on Bristol Road in close proximity to one of the fast-food kitchen vents on the road, with participants noting it frequently being in use late at night. Consequently, a possible explanation for the overnight peak in F1 at H1 is the infiltration of particles generated from commercial cooking activities which are common along Bristol Road. Cooking periods recorded by occupants had no clear connection to PM concentrations or source contributions. Each bedroom was located on the floor above the kitchen, and the houses had closed floor plans, potentially limiting the dispersion of PM from these activities to be detected as they happened by the LCS. Research conducted into PM generated from cooking has found the particles to generally be in the UFP and PM_2.5_ size ranges^16^. The UFP size range is below the lower detection limit of the LCS, however the greater contribution of F1 to the PM_1_ and PM_2.5_ size fractions support the possibility of this factor being associated with cooking activities. At H1, F1 appears to be associated with the restaurants on the Bristol Road, so this source, at least in part, originates from an external cooking source and was considered as an outdoor source, thought indoor cooking activities likely also contributed to this factors profile in H2 and H3 which were further from the restaurant sources. This shows that when this methodology is applied across multiple households where the environment is not the same, sources identified by the NMF which have the same PNSD, can have very different profiles in these different environments.

Finally, the outcomes of the NMF analysis were compared with results using the established Positive Matrix Factorisation (PMF) method (results not included). The factors formed from both methods presented great similarity (factors describing similar sources presented Pearson correlation coefficient r > 0.84), pointing that the NMF can be used as a reliable alternative to the PMF.

### Influence of indoor and outdoor sources

The average I/O source contribution ratios calculated from the NMF results ranged from 0.08 (H1, PM_1_) up to 4.93 (H2, PM_10_). The findings of increasing indoor source contributions to larger PM size fractions aligns with findings of a previous study^13^, in which LCS source apportionment was utilized cross different rooms within one house. Here, I/O effects decreased with PM size. The smaller PM_1_ size fraction was recorded in the highest concentration and found to be mostly made up of outdoor factors, in an office room which had the greatest natural ventilation. However, indoor source contributions increased for larger PM_2.5_ and PM_10_ size fractions^13^. Across literature, calculated I/O ratios are highly varied, with some finding ratios < 1^13,31,32^, others > 1^24,34^. When ratios above 1 have been reported, there have been particular indoor sources noted, such as wood-burning appliances^24^, or increased indoor activity levels^23^. This is supported by the finding that (with the exception of PM_2.5_ and PM_10_ at H2) the I/O ratios were lower than average during periods when the bedrooms were unoccupied, showing outdoor source influence to be greater when indoor activity was lowest.

Compared with the I/O ratios modelled in the similar indoor low-cost PM source apportionment study by Bousiotis et al.^13^ which were all < 1, the average ratios found across the houses in this paper are much higher for the larger particle sizes (PM_2.5_ and PM_10)_. It is likely that the ventilation differences between the houses studied contributed to this. In the aforementioned study, the windows had trickle vents, allowing ambient air to continuously enter the rooms even if windows and doors were shut. These did not feature on the windows in the present study, meaning that along with the very infrequent window opening rates (particularly in H2 which saw the highest PM concentrations and I/O ratios), there was very little ambient air ventilation in the bedrooms studied. Resultingly, there was less opportunity for outdoor air to enter the bedrooms, but also for indoor air and PM to disperse out of the rooms.

The lowest I/O ratios were determined at H1, followed by H3 and then H2, indicating outdoor sources to be least influential at H2—the furthest from the traffic and restaurant sources densely packed on Bristol Road upon which H1 is located. However, whilst this generally supports the hypothesis that outdoor sources would be more influential at the house in closest proximity to significant outdoor sources, the strength of this evidence would be improved through comparing I/O ratios when the houses are empty for a prolonged period at the same time. Unfortunately, during the monitoring campaign, no such period occurred for direct comparison of the influence of outdoor sources within each house.

### Study limitations

With the lower detection limit for particle diameter of the Alphasense OPC-N3’s being 0.35 μm, particles smaller than this were not recorded by the LCS. Although unlikely to highly influence found PM mass concentrations because of their extremely small masses, it means the LCS missed key particles from both indoor or outdoor sources which could affect the source apportionment which is based upon PNSD. The short duration of the monitoring campaign meant robust comparisons of I/O ratios during empty periods could not be undertaken, with no simultaneous empty periods across the houses. Within each house, 1 bedroom was monitored; previous studies have found this room to have the highest associated PM concentrations due to the presence of soft-furnishings and activities^13^ and so these rooms likely record higher PM concentrations than averaged throughout the house. However, as this room is where the occupants spent most of their time whilst in the house, it is a good room to monitor to understand concentrations occupants may be exposed to. Whilst the preformatted time activity diaries were generally well completed, by nature of their design they provided coarser temporal resolution than had sensors or direct observation been used to record human activity. Despite this, with the activity data gathered, this has shown that relatively minimal participant burden and costs are needed to capture activity within the house which influenced PM concentrations.

## Conclusions

To our knowledge, this is the first study to apply LCS source apportionment techniques to investigate and compare PM concentrations and source contributions across multiple households. The three LCS were calibrated against a research grade instrument, with which they were in very good agreement. Following a 2-week monitoring campaign, it was found that PM concentrations in all three houses were greater, more variable and significantly different to ambient concentrations recorded at a nearby monitoring site. It was also found that at one house the WHO 24-h guideline limit for PM_2.5_ was breached on 9 individual days throughout the campaign. These findings highlight the importance on monitoring indoor air pollution to provide data to improve pollution exposure estimates, as whilst people may live in areas with acceptable ambient air quality, they may be exposed to unhealth concentrations in their own homes. Concentrations were also seen to be significantly different between houses in the same residential area, emphasising the heterogeneity of indoor environments and demonstrating that monitoring just one indoor location is unlikely to be representative of concentrations across a residential area. Source apportionment, using NMF, was applied to all three houses simultaneously to look for common sources between houses. The applied NMF methodology was highly successful at accurately modelling indoor PM concentrations across all of the houses, which can help to improve pollution exposure estimates at a relatively low cost, with the highest agreement between modelled and measured PM concentrations for PM_10_. It could also provide insight to the source influences across multiple households, with factors linked with indoor activity were clearly identified using information from time activity diaries. Indoor factors generally had the greatest influence on larger particle sizes, with average I/O ratio across the houses of 3.66 for PM_10_, 1.02 for PM_2.5_ and 0.30 for PM_1_, supporting the hypothesis that PM_10_ would be most greatly influenced by indoor activity. Source identification however could not be confidently specified beyond indoors or outdoors. Future studies may consider applying this methodology to an extended monitoring period to evaluate seasonal source variations, which may help to further narrow down the source identification. Studies could also scale up the methodology to investigate source variability across larger geographical areas.

## Methods

This project received ethical approval from the University of Birmingham Science, Technology, Engineering and Mathematics (STEM) ethics committee, number ERN_3249. Informed consent was obtained from each participant, and all methods were carried in accordance with relevant guidelines and regulations.

To compare and investigate the heterogeneity of air quality across households within the same residential area, a monitoring campaign was conducted across three student households using LCS, to obtain particle number size distributions (PNSD) and PM concentrations. A non-negative matrix factorisation (NMF) source apportionment technique was applied to investigate the sources of PM within the households and compare how these sources differed between the houses.

### Monitoring campaign

**Fig. 6:**
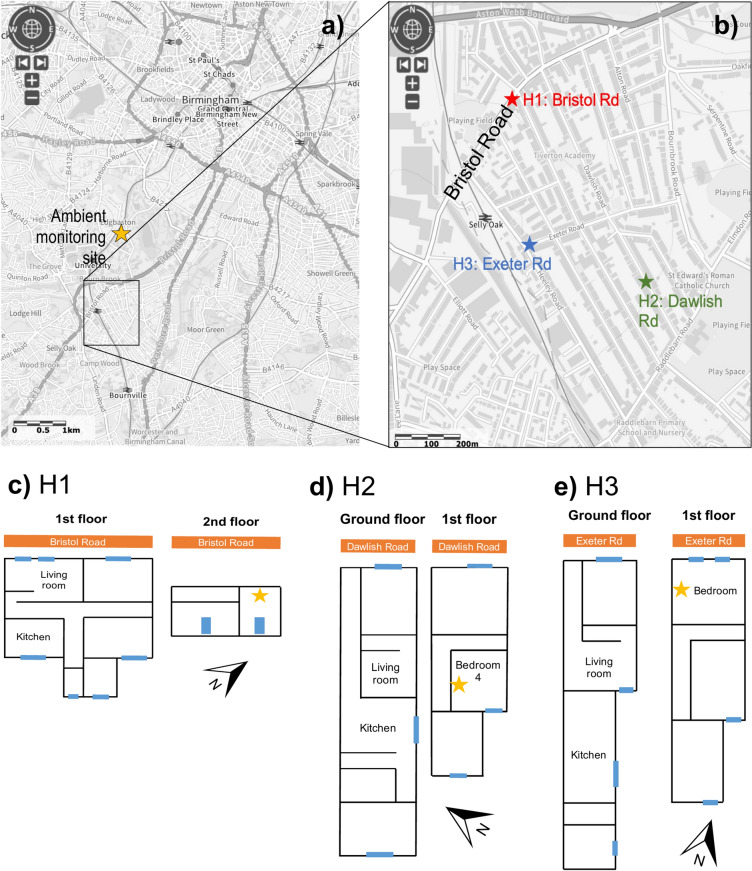
(**a**) map showing the monitored residential area in relation to Birmingham city centre and nearby Birmingham Air Quality Supersite, and (**b**) location of the three monitored houses within the Selly Oak residential area of Birmingham, UK. Floor plans (approximate, not to scale) are given in (**c**) H1, (**d**) H2 and (**e**) H3. Blue boxes are windows (roof windows on H1 2nd floor). Star denotes LCS location in the monitored bedroom. The map in (**a**) is from the Multi-Agency Geographic Information for the Countryside, which uses Ordnance Survey data with Crown Copyright and database rights 2024. Ordnance Survey AC0000805307.

*Study location* The three households studied are within the Selly Oak urban residential area (52.4409, − 1.9386), which is approximately 4 km southwest of Birmingham (UK) city centre. The houses are within 1 km of each other (Fig. 6). Each house is a student household, with H1 occupied by four students, located on Bristol Road (B384) above a fast-food restaurant whose kitchen vent is situated adjacent to the house. H2 is on Dawlish Road, and H3 on Exeter Road, both more minor roads, and occupied by five and four students respectively. There are no smokers in any of the houses. All houses have central gas heating, and a gas-fired combined hob and oven. Whilst H3 has an overhead extractor fan typically used during cooking times, H1 has no extractor fan, and H2 has an axial extractor on the kitchen wall. The monitoring period lasted 2 weeks, from March 8, 2022 until March 22, 2022.

*Instrumentation* In each house, one Alphasense OPC-N3 sensor was deployed in a bedroom (all with carpeted flooring) for the full study period duration (see approximate house layouts in Fig. 6c–e). This optical particle counter measures light scattering from particles ranging from 0.35 to 40 μm (spherical equivalent size), which get assigned to 1 of 24 size bins, at a count rate up to 10,000 particles per second^35^. Particle number size distribution (PNSD) data is recorded and used to internally calculate PM_1_, PM_2.5_ and PM_10_ concentrations, collected at a 10-s resolution. 10-min measurement averages were used for analysis, and there was 100% data availability for the monitoring period. Meteorological data and ambient PM_2.5_ and PM_10_ concentrations during the study period were obtained from the nearby Birmingham Air Quality Supersite (BAQS), which is an urban background site approximately 1.5 km north of the study area (see Fig. 6). The BAQS PM data was collected using a regulatory grade Palas FIDAS monitor.

*Activity data* was collected throughout the study period by household members using a formatted activity diary, to understand activity within the households. Data was collected for activities in the houses including occupancy levels, cooking times and types, and other activities which may influence the air quality, with one diary for the house in general and one for the bedroom in which the OPCs were located. Participants were not expected to change their usual routine, other than to fill out the activity diaries to the best of their ability each day. These were pre-formatted, as previous studies have found this eases completion and minimises participant burden, encouraging better participant engagement^36,37^.

### Calibration

A review of research into indoor air quality using LCS has found many studies failed to perform calibration or validation of the sensors, hindering confidence in results^38^*.* It has been documented that the Alphasense OPCs are affected by PM hygroscopic effects when ambient RH is high, and can overestimate PM mass concentrations^29^. For calibration, the OPCs were collocated in an indoor environment for 2-days alongside a TSI-3330 Optical Particle Sizer (which measures particle sizes 0.3–10 μm). It is noted that this calibrated research grade instrument does have reported limitations, with Rivas et al.^39^ finding non-existent peaks and zero concentrations in very clean conditions. Nevertheless, for calibration, the OPC data was averaged to a 1-min time resolution for consistency with the TSI sampling rate.

### Data analysis

To investigate the sources and their relative contribution to PM levels between the houses, non-negative matrix factorisation (NMF) was employed. NMF data analysis is a data dimensionality reduction technique, similar to positive matrix factorisation (PMF), which is a widely used source apportionment method, frequently applied to both regulatory grade and LCS outdoor air pollution studies^40–43^ and recently in a LCS indoor study^13^.

NMF was first introduced by Lee and Seung^44^. Subsequently, NMF has been applied, and used in aerosol source apportionment of LCS data in Delhi, India^45^.

For this study, the default NMF algorithm method used is based on that employed by Brunet et al.^46^. The algorithm is an iterative process which computes an approximation A ~ WH, with both W and H being non-negative matrices. Matrix A contains PNSD data across N columns of bin sizes for M rows which here are 10-min averaged timesteps, chosen to capture dynamic indoor conditions. The desired rank k is set and initial W and H matrices iteratively updated: Matrix W is of the size N x k, with each of the k columns defining a factor, and each row containing the contribution of that factor for each given timestep. Matrix H is of the size k x M, where each of the M columns represents the average particle count per bin size, giving the average PNSD and PM concentrations for each factor (rows). The factorisation A ~ WH is then used to group the M samples into k clusters, in this case referred to as factors. The non-negativity requirement of NMF provides algorithmic complexities, however this allows for more intuitive data decomposition into interpretable factors^46^. As a descriptive model, there is no objective criterion for the optimum value of k to be used; key to determining which rank of k to choose is determining if clusters can be physically interpreted as distinct, identifiable source profiles^41,46^. To comply with this the following criteria were considered in the choice of the number of factors:

- The factors should be interpretable (factors should be understood and associated with sources, activities etc.)
- The factors should be unique (factors should be significantly different)
- The factors should have a significant effect on the variables studied (solutions with factors with almost zero concentrations on all the variables inputted were not considered)
- The reduction (as much as possible) of the unexplained variation of the variables

Unlike the PMF, the NMF does not have a specified method which determines when convergence is reached. Furthermore, the measurements’ uncertainty is not an input but is calculated instead as the model tries to converge the matrices formed with the measurements provided. To address these limitations the resampling method was chosen for this study. While resampling is a computationally intensive work, advancements in the computational power have made such approaches trivial for relatively small datasets as the one used for this study. Thus, several runs with a great number of resampling (100 resamples) and iterations (700 iterations per run) were done. With that, convergence was assured while the relative error was kept to a minimum (< 1%).

To estimate PM concentrations, elements within the H matrix were multiplied by elements of the W matrix. The H matrix denotes the average mass particle concentration for each factor, whilst the W matrix contains the normalised (average of 1) contribution of each factor at each given timestep. Thereby by multiplying these matrices, an estimate of the PM concentration of each factor for every 10-min timestep is obtained. It is noted this method may carry inaccuracies, with PM concentrations calculated according to an average across all houses, however it allows for comparison of the contribution of each factor to PM concentrations between the houses in the study.

To interpret the factors returned by the NMF, analysis of each factor’s PNSD, diurnal variation, and relative contributions to PM concentrations was undertaken. Information from participant time-activity diaries and meteorological conditions was also used to further understanding of the factors. Together, this analysis was used to identify the source associated with each factor. The NMF analysis was undertaken using the NMF package for R^47^, and further analysis was performed using the Openair package for R^48^.

Kruskal–Wallis rank sum tests and post-hoc Dunn tests (with the Bonferroni p-value adjustment method applied to limit type-1 error rates) were performed to determine whether there were statistically significant differences between PM concentrations measured at each location (the three houses, and where applicable ambient). Non-parametric data analysis tests were performed, as the PM concentration data was highly positively skewed and violated normality assumptions even after logarithmic and power transformations were applied.

## Supplementary Information

Supplementary Information.

## Data Availability

Data supporting this publication are openly available from the UBIRA eData repository 10.25500/edata.bham.00001018, which is a public repository that meets appropriate standards of archiving, citation and curation.
