# Supplementary material for: Using low-cost sensors to assess common air pollution sources across multiple residences
Source: Sci Rep. 2025 Jan 13;15:1803. doi: 10.1038/s41598-025-85985-1 (PMC11729851; doi:10.1038/s41598-025-85985-1)
Supplement: Supplementary file 1 — Supplementary Information. [file 41598_2025_85985_MOESM1_ESM.docx]

**Supplementary Materials**

**Using low-cost sensors to assess common air pollution sources across multiple residences**

Catrin J. Rathbone, Dimitrios Bousiotis, Owain Rose and Francis D. Pope


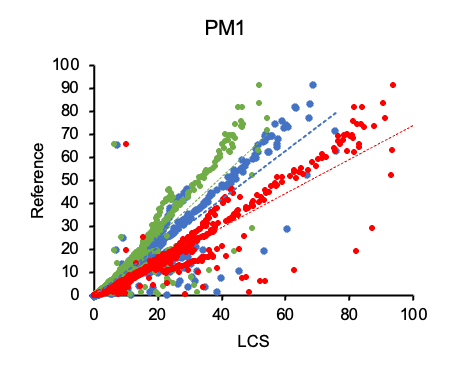


*r* = 0.946

*r* = 0.940

*r* = 0.956

a)


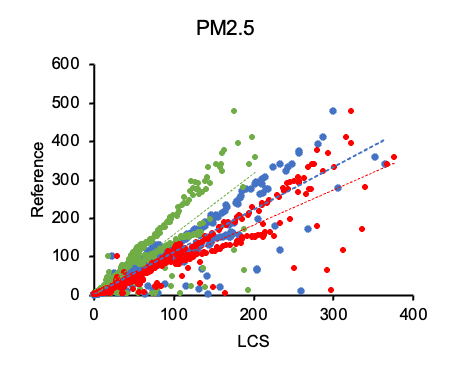


*r* = 0.947

*r* = 0.924

*r* = 0.950

b)


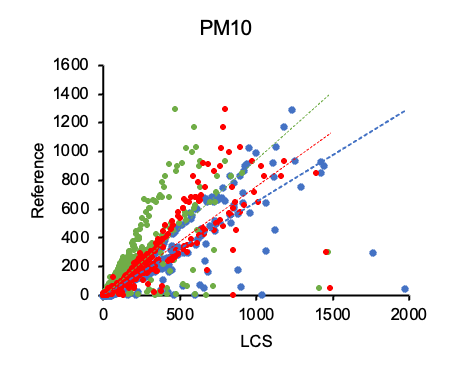


*r* = 0.889

*r* = 0.799

*r* = 0.902

c)


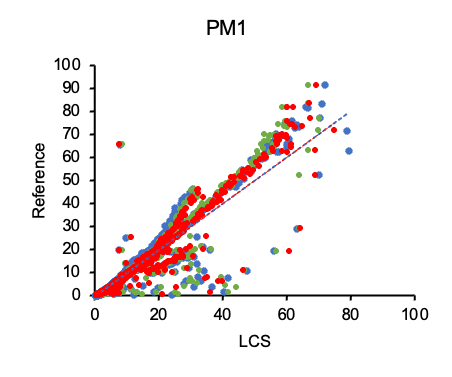


*r* = 0.945

*r* = 0.939

*r* = 0.955

d)


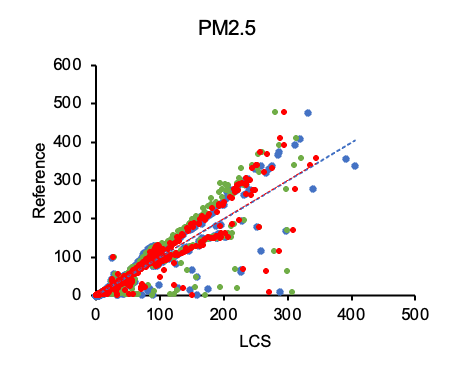


*r* = 0.946

*r* = 0.923

*r* = 0.950

e)


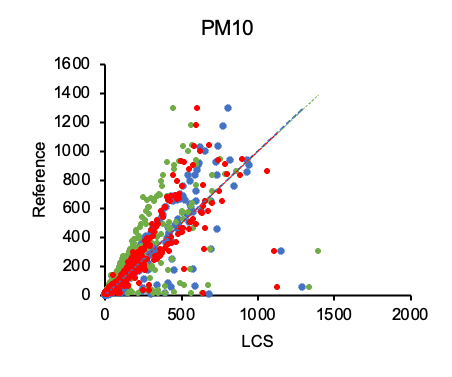


*r* = 0.889

*r* = 0.799

*r* = 0.902

f)

**Legend**

OPC1 Linear (OPC1)

OPC2 Linear (OPC2)

OPC3 Linear (OPC3)

**Fig S1:** Scatter plots showing correlation between PM concentrations at 1-minute resolution between the low-cost sensors (LCS) and reference instrument used, for each PM size fraction before calibration (a-c) and after calibration (e-f). Line of best fit and correlation coefficient denoted for each individual OPC sensor against the reference instrument. See legend for colour code of each OPC.

(a)

(b)

(c)

**Fig S2:** Correlation plots of 10-minute average NMF modelled PM concentrations against LCS measured PM concentrations for all houses (see key for colour code), for a) PM_1_, b) PM_2.5_ and c) PM_10_.

**Fig S3**: Particle number size distribution (PNSD) profiles for each factor.

**Table S1:** Summary statistics for PM_1_, PM_2.5_ and PM_10_ concentrations at each site over the 2-week monitoring period. Mean, standard deviation (s.d.), median and interquartile range (IQR) given. Same upper-case letters indicate no significant differences (p > 0.05) between sites, according to Kruskal-Wallis post-hoc Dunn test results.

| **Site** | **Mean** | **s.d.** | **Median** | **IQR** |
| --- | --- | --- | --- | --- |
| *PM_1_* | | | | |
| H1 | 4.19 | 6.13 | 2.20 | (1.09 – 4.54)^A^ |
| H2 | 4.90 | 7.94 | 1.98 | (1.05 – 4.74)^A^ |
| H3 | 1.83 | 2.30 | 1.00 | (0.59 – 1.97)^B^ |
| *PM_2.5_* | | | | |
| H1 | 10.55 | 17.02 | 5.08 | (2.38 – 10.4)^A^ |
| H2 | 19.87 | 41.62 | 6.52 | (3.40 – 16.4)^B^ |
| H3 | 5.47 | 6.83 | 3.13 | (1.84 – 6.16)^C^ |
| Ambient | 4.82 | 3.28 | 4.07 | (2.98 - 5.55)^D^ |
| *PM_10_* | | | | |
| H1 | 15.79 | 29.16 | 7.18 | (2.88 – 15.3)^A^ |
| H2 | 30.55 | 93.19 | 7.58 | (3.51 – 18.3)^B^ |
| H3 | 10.04 | 20.22 | 5.11 | (2.64 – 10.7)^C^ |
| Ambient | 6.93 | 4.36 | 5.94 | (4.27 - 8.05)^D^ |

**Table S2**: Pearson correlation coefficients between the contributions of the factors from the NMF analysis

|  | **F1** | **F2** | **F3** | **F4** | **F5** |
| --- | --- | --- | --- | --- | --- |
| **F1** | - | 0.68 | 0.33 | 0.20 | 0.62 |
| **F2** | 0.68 | - | 0.39 | 0.17 | 0.29 |
| **F3** | 0.33 | 0.39 | - | 0.77 | 0.12 |
| **F4** | 0.20 | 0.17 | 0.77 | - | 0.06 |
| **F5** | 0.62 | 0.29 | 0.12 | 0.06 | - |

**Table S3:** The average PM_1_, PM_2.5_ and PM_10_ concentrations ($\mu$g m^-3^) modelled by the NMF methodology for each household is given in the ‘Total’ row, with the average G-contribution of each factor (F1-5) found for each PM size fraction at each house detailed.

|  | **H1** | | | **H2** | | | **H3** | | |
| --- | --- | --- | --- | --- | --- | --- | --- | --- | --- |
|  | PM_1_ | PM_2.5_ | PM_10_ | PM_1_ | PM_2.5_ | PM_10_ | PM_1_ | PM_2.5_ | PM_10_ |
| *Total* | *5.21* | *10.62* | *20.32* | *4.28* | *13.53* | *33.07* | *1.75* | *4.08* | *15.07* |
| F1 | 1.88 | 2.76 | 2.63 | 0.89 | 1.32 | 1.25 | 0.37 | 0.55 | 0.53 |
| F2 | 1.73 | 4.32 | 5.43 | 1.14 | 2.85 | 3.58 | 0.53 | 1.33 | 1.66 |
| F3 | 0.30 | 1.77 | 2.51 | 1.27 | 7.47 | 10.63 | 0.32 | 1.86 | 2.64 |
| F4 | 0.08 | 0.55 | 8.65 | 0.16 | 1.08 | 16.86 | 0.09 | 0.63 | 9.84 |
| F5 | 1.22 | 1.21 | 1.10 | 0.82 | 0.82 | 0.74 | 0.44 | 0.44 | 0.40 |
